# Announcing the Tumor Immunology and Biological Cancer Therapy section (edited by iSBTc) of the *Journal of Translational Medicine*

**DOI:** 10.1186/1479-5876-7-80

**Published:** 2009-09-17

**Authors:** Pedro Romero, Bernard A Fox, iSBTc Executive Office

**Affiliations:** 1 Division of Clinical Onco-Immunology, Ludwig Institute for Cancer Research, Lausanne branch, University Hospital (CHUV), Lausanne, CH-1011, Switzerland; 2 Department of Molecular Microbiology and Immunology, Oregon Health and Science University and Robert W Franz Cancer Research Center, Earle A Chiles Research Institute, Portland, OR, USA; 3 Publications, International Society for Biological Therapy of Cancer, Milwaukee, WI, USA

## Editorial

The International Society for Biological Therapy of Cancer (iSBTc) and BioMed Central are proud and delighted to announce their collaboration in editing a section of the *Journal of Translational Medicine *devoted to promoting the field of Tumor Immunology and Biological Cancer Therapy.

## Addressing a need for a dedicated forum on cancer immunology and immunotherapy

We are convinced that we are living in exciting times for this emerging field. Major and multiple discoveries in immunology have been expanding the frontiers of our understanding of immunity at an ever increasing pace. These discoveries include a long list of cytokines, the elucidation of the structural basis of antigen recognition by the cells of the adaptive immune system - the B and T lymphocytes -, the identification of dendritic cells, the dissection of the mechanisms of thymic selection of the mature T cell repertoire, the outlining of T and B cell differentiation pathways, the mapping of crucial checkpoints along the pathways of adaptive immune responses, the conclusive demonstration of immunosurveillance against tumors, and the molecular links between innate and adaptive immune response mechanisms. In parallel, the translation of major insights made in animal models to the clinics has also picked up momentum. Translational research has gained a respectable place among academic, industry and government institutions alike. Recent years have brought a busy and exciting dialog between the bench, the bedside and back to the bench. A large number of novel therapies based on the immunobiology of tumors have been or are currently being tested in the clinics. As well, sophisticated preclinical models of major tumor types are generated at an astonishing speed to address major questions of basic and applied cancer research. The community as a whole is now oriented toward the prospect of identifying combination anti-cancer therapies that may reach unmatched clinical efficacy. Such levels of activity require appropriate vehicles and means to share new knowledge and to catalyze the development of the field.

The boundless possibilities offered by a rapidly published, open-access, online communication of results and exchange of opinions are potent tools to most efficiently achieve these tasks. Thus, this section of the *Journal of Translational Medicine *provides a venue for publication of original research articles, literature reviews, opinion/position papers and a forum to discuss the hot issues in Tumor Immunology and Biological Cancer Therapy research. On behalf of the International Society for Biological Therapy of Cancer, we would like to invite you to submit your work, your thoughts and/or to engage in informal discussions. We welcome you and look forward to your contributions.

Pedro Romero, MD

Section Editor, Tumor Immunology and Biological Cancer Therapy (edited by iSBTc)

Bernard A. Fox, PhD

President

International Society for Biological Therapy of Cancer

